# A Narrative Review on Mobile Health (mHealth) App for Stroke Care and Rehabilitation Intervention for Malaysia

**DOI:** 10.21315/mjms-03-2025-154

**Published:** 2025-06-30

**Authors:** Tuan Siti Mastazliha Long Tuan Kechik, Kamarul Imran Musa, Jafri Malin Abdullah, Sureshkumar Kamalakannan, Norsima Nazifah Sidek, Nurfaten Hamzah, Muhammad Hafiz Hanafi

**Affiliations:** 1 Department of Neurosciences, School of Medical Sciences, Health Campus, Universiti Sains Malaysia, Kelantan, Malaysia; 2 Department of Community Medicine, School of Medical Sciences, Health Campus, Universiti Sains Malaysia, Kelantan, Malaysia; 3 Brain Behaviour Cluster, School of Medical Sciences, Health Campus, Universiti Sains Malaysia, Kelantan, Malaysia; 4 Department of Social Work, Education, and Community Well-being, Northumbria University, Coach Lane Campus, Newcastle upon Tyne, United Kingdom; 5 Division of Care in Long Term Conditions, King’s College London, London, United Kingdom; 6 Clinical Research Centre, Hospital Sultanah Nur Zahirah, Ministry of Health Malaysia, Terengganu, Malaysia; 7 Rehabilitation Medicine Unit, School of Medical Sciences, Health Campus, Universiti Sains Malaysia, Kelantan, Malaysia

**Keywords:** mHealth, stroke mobile application, stroke rehabilitation, stroke caregivers, caregiver burden

## Abstract

The 2019 COVID-19 pandemic exposed significant challenges in global healthcare. In Malaysia, limitations in stroke care and the lack of digital interventions highlighted gaps in supporting the stroke community, increasing caregiver burdens and undermining the nation’s stroke care system. The study is conducted to explore evidences about the digital health in stroke care and rehabilitation, especially for home-based care in Malaysia, including the current status and the availability of intervention modules. The literature search for this narrative review was conducted across databases such as PubMed, Scopus, Google Scholar, and others. Approximately 250 articles were identified through a combination of database searches and manual selection using keywords such as “stroke rehabilitation,” “digital health,” “mHealth,” “caregiver burden,” and “Malaysia”. After screening 127 articles based on their titles and abstracts, 80 full-text articles were assessed for relevance to the review’s inclusion criteria. Ultimately, 58 studies (research articles and website sources) were included in the final review, focusing on stroke rehabilitation, caregiver burden, and digital health interventions, particularly mobile health (mHealth) solutions. The results are presented in summative paragraphs of reviews complementing subtopics in order to reach final conclusion and future directions. The subtopics are: i) Understanding stroke and caregiver’s burden; ii) Stroke in global digital health; iii) Stroke intervention modules for web-based and smartphone applications; and iv) Malaysia, mobile health (mHealth) and stroke intervention module. Evidence suggests that Malaysia still falls short in meeting the needs of the stroke community, and that stroke care must be made available through digital means.

## Introduction

Stroke has consistently remained a leading cause of adult disability, with devastating consequences for survivors and their caregivers (1). While stroke management has advanced significantly over time, statistical data continue to report increasing trends in the population of stroke survivors, many of whom live with varying neurological and functional deficits (1). Despite these challenges, the field of stroke research has expanded significantly, contributing to an improved understanding of stroke causation and enabling the development of novel treatments, advanced technologies, and innovative interventions. These developments hold promise for a better future in stroke care and rehabilitation (2).

According to the Global Burden of Diseases, Injuries, and Risk Factors Study (GBD) 2019, stroke remains a top contributor to global mortality, ranking among the top three causes of total deaths across 204 countries and territories, including Malaysia (3). While the global trend of stroke-related mortality showed a decline of 38% from 2010 to 2019, this pattern contrasts sharply with Malaysia’s situation, where stroke deaths have remained alarmingly high. Data from the World Health Organization (WHO) in 2018 indicated that stroke deaths in Malaysia accounted for 13,799 cases or 9.8% of total deaths, with an age-adjusted death rate of 62.64 per 100,000 population due to cerebrovascular diseases. These figures positioned stroke as the third leading cause of death in the country (3–5). Additionally, preliminary data from the project “Monitoring Stroke Burden in Malaysia 2017,” published in 2019, revealed an average of 92 stroke admissions daily across Malaysia’s healthcare facilities, with nearly 32 deaths per day. Alarmingly, stroke is projected to become the second leading cause of mortality in Malaysia by 2040 (6). These statistics highlight the pressing need for effective stroke care strategies to mitigate the growing burden of this disease.

The global healthcare sector, including Malaysia, continues to invest in strategies and technologies aimed at improving stroke treatment, rehabilitation, and monitoring (7, 8). Extensive research has been conducted worldwide on the burdens faced by stroke patients, survivors, and their caregivers, with findings consistently highlighting significant psychological, economic, and physical challenges (9, 10). For example, Firmawati et al. (11) emphasise the role of mobile health applications in supporting family caregivers in recurrent stroke prevention, which can mitigate the psychological and physical toll experienced by caregivers. Similarly, Bonura et al. (12) provide a comprehensive overview of smartphone applications that facilitate stroke management, suggesting these tools can support both survivors and caregivers in navigating post-stroke challenges. Malaysia’s digital health system status, particularly concerning stroke care, remains unexplored. To address this gap, the objective of this paper is to review and assess matters regarding stroke care, caregivers’ burden and the current status of the nation’s digital health intervention modules. It is important to know how far we have come in order to reach an advanced level for our systematic healthcare services.

The COVID-19 pandemic further amplified the urgency for alternative care strategies. During the pandemic, the Movement Control Order (MCO) imposed in Malaysia created substantial barriers to traditional healthcare services, including hospital-based stroke care and rehabilitation. This unprecedented situation shifted the focus toward home-based stroke management and underscored the potential of digital health interventions as a viable solution. Digital health tools such as mobile health (mHealth) applications have been identified as effective platforms for extending stroke care beyond clinical settings, facilitating remote monitoring, and providing ongoing support (12, 13). While the pandemic served as the initial catalyst for this exploration, the need for remote and flexible stroke care persists beyond the pandemic context.

As the digital health landscape evolves globally, there is an increasing need to assess Malaysia’s position in leveraging digital health interventions for stroke care (14). Mobile health (mHealth) applications and other digital health technologies hold immense potential for addressing the gaps in stroke rehabilitation and monitoring, particularly in home-based settings (15). Yet, the adoption and implementation of these technologies in Malaysia remain inadequately explored, presenting a critical gap in healthcare delivery.

This review aims to explore the role of digital health, with a focus on mHealth applications, in supporting stroke care and rehabilitation, particularly in home-based settings. While inspired by the challenges faced during the pandemic, this study seeks to provide insights that remain relevant in addressing the broader, long-term needs of stroke care within the Malaysian healthcare context and beyond. By understanding how digital health interventions can enhance stroke management, this work aims to contribute to the development of more resilient and effective healthcare strategies for stroke survivors.

## Methods

**Figure 1:**
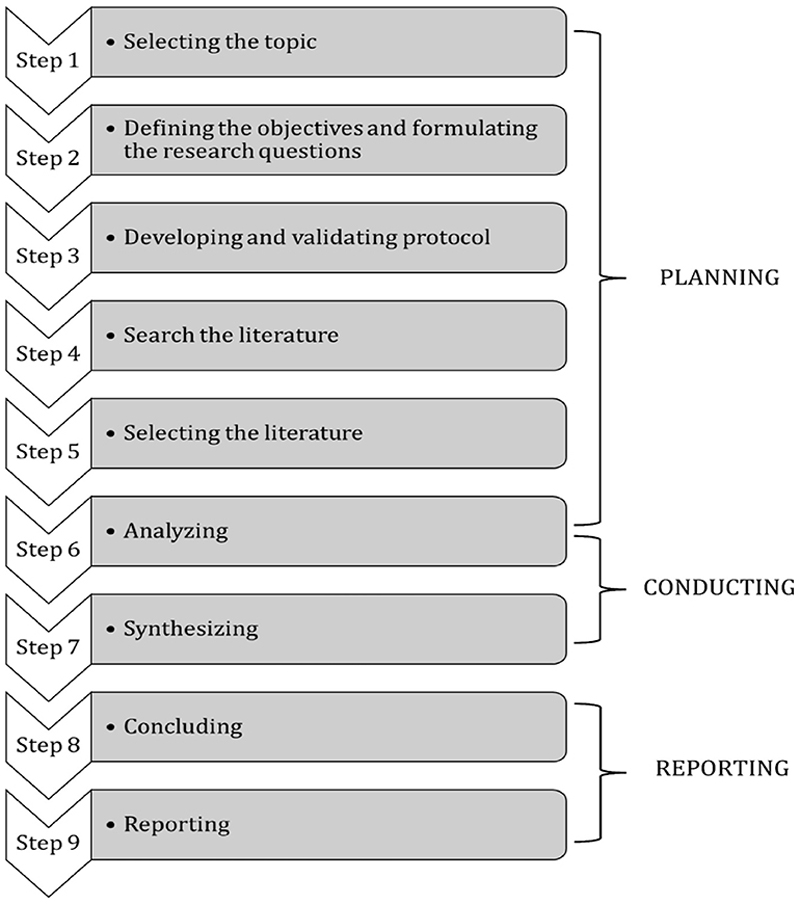
Illustration of the phases and steps of a narrative literature review, adapted from Juntunen and Lehenkari (16)

A narrative literature review was conducted to address the gap in the availability of mHealth for the stroke community in Malaysia, including stroke care, caregiver’s burden and rehabilitation intervention. The purpose of using a narrative literature review in this study was to provide an up-to-date account of what is already known about the digitalisation of stroke care and rehabilitation intervention for Malaysia exclusively, but including global information as well as reference reasons. Three phases and nine steps of a narrative literature review were adopted to answer the review question (16). An illustration (Figure 1) was adapted from Juntunen and Lehenkari (16) and referred to as procedure guide, which depicts the phases and steps of a narrative literature review followed in this study:

### Step 1: Selecting the Topic

The topic selected for this study is mHealth app for stroke care and rehabilitation intervention for Malaysia.

### Step 2: Defining the Objectives and Formulating the Research Questions

The objective of this review is to explore the availability of digital health interventions, including mHealth applications, in stroke rehabilitation, with a focus on reducing the caregiver burden for stroke community in Malaysia. This study aimed to address the following review question:

“Does Malaysia currently have a digital mHealth intervention in addressing the needs for stroke care and rehabilitation?”

### Step 3: Developing and Validating a Review Protocol

This step is comparable to research design in empirical research, and it contains a pre-set plan for how researchers aim to conduct all other steps of the research process. In this context, the first author conceptualised the study. Validation of the protocol was performed by every other author involved. All authors contributed equally in finalising the manuscript.

### Step 4: Search the Literature

Purposive sampling was applied using Google Scholar and Resipotary@USM. Additionally, academic journals from electronic databases such as PubMed, Frontiers, SCOPUS, Google Scholar, and ScienceDirect, as well as official organisational websites, were accessed through ad-hoc searches conducted via the Google search engine from January 2021 to December 2023. Manual searches of cross-references and textbooks were also conducted concurrently. Any type of literature and editorials, including official government reports published online and offline, are included (17–19).

The keywords used for the searches are listed in Table 1. Search engines or websites were run using the Boolean operators to help combine keywords effectively as follows: i) only main keywords; ii) main keywords with any keyword’s inclusion; and iii) combination of keywords (multiple main’s “OR”/”AND” with inclusion’s). For example, “Stroke AND mHealth AND Malaysia” and “Mobile Health Application AND Stroke”.

### Step 5: Selecting the Literature

All authors agreed on the inclusion and exclusion criteria of this study. This study included published articles on online databases journals and editorials provided by official health organisational websites between 2013 and 2023 to ensure the inclusion of current technologies and interventions (20, 21). Citations of articles, journals and reports were tracked to locate additional studies and information when exploring diverse literature in which terminology is used inconsistently. Any older publications were selected as background main points and references related. This study excluded research unrelated to stroke or digital health, as well as articles and editorials lacking proper citations or accreditation from official sources, authors, or websites.

### Steps 6–8: Analysing, Synthesising and Concluding

Given the exploratory nature of this narrative review, a manual selection process was used to allow for flexibility in identifying relevant literature across various sources and formats. This approach enabled a broader exploration of stroke rehabilitation and caregiver burden in digital health contexts, considering the limitations on the availability of publications on this topic, especially those covering Malaysia. Studies were included if they focused on stroke rehabilitation through digital health interventions, addressed caregiver burden, or focused on mHealth interventions, particularly in Malaysia or similar settings.

The literature search for this narrative review was conducted using a combination of academic databases such as PubMed and Scopus, along with Google Scholar. Additionally, other sources were identified through ad-hoc searches performed using Google, which may have included studies from various databases and repositories not explicitly tracked during the search process. Approximately 250 articles were identified through a combination of database searches and manual selection. After screening 127 articles based on their titles and abstracts, 80 full-text articles were assessed for relevance to the review’s inclusion criteria. Ultimately, 58 studies were included in this final review, focusing on stroke rehabilitation, caregiver burden, and digital health interventions, particularly mHealth solutions.

A narrative (hermeneutics) approach to summarise and emphasise key contributions is used. The data presented in this review are the same as those found in the source articles; no data synthesis or meta-analysis has been performed. Data were extracted based on their relevance to the topic instead of implementing a systematic approach to paper selection. Although the main focus is to address Malaysia’s epidemiology and territorial issues, other countries are reviewed as references and for comparative reasons (17–19).

### Step 9: Reporting

The structure of this literature review includes the title, abstract, introduction, methods, results (presented as subtopics), discussion and conclusion with a list of references (16).

## Results

### Understanding Stroke and Caregivers Burden

Stroke rehabilitation involves a collaborative team effort that includes patients, their families (primary caregivers) and friends, secondary caregivers, physicians, neurologists, nurses, physical and occupational therapists, speech-language pathologists, recreation therapists, psychologists, nutritionists, social workers, and community support personnel. Communication and cooperation among these specialists are critical for post-stroke therapy and recovery success (2, 22). Stroke survivors should continue with ongoing rehabilitation following a stroke after leaving the hospital. The specialised services can be accessed via facility-based outpatient services and in-home rehabilitation services (23). Age and initial stroke severity are the two most powerful predictors in determining the functional recovery and discharge plan of stroke patients. Based on stroke severity, an optimal stroke rehabilitation programme could be determined, all based on the individual patients’ specific needs. For severe stroke patients, it is recommended to undergo in-hospital intensive interdisciplinary stroke rehabilitation, and for moderate stroke patients, it is recommended to commit to hospital programmes that are usually the most comprehensive, provide the greatest intensity of therapy, and generally have optimal medical coverage (23).

Meanwhile, mild stroke patients can generally be managed directly in the community as long as it is feasible, and this includes home care rehabilitation. As advocated by Edmonds and Peat (24) decades ago, the outpatient and home care rehabilitation approach is multidisciplinary in nature as it takes place where the individual lives and existing community resources are used with the full involvement of family members or caregivers. The advantages of home care approach are the potential for more involvement between patients and family members or caregivers to a greater extent in the stroke patients’ health care (24, 25). Ever since then, more reports have been published proving the benefits and effects of localising stroke rehabilitation at home and within the community. The community empowerment goals include prevention, delay, and management of stroke conditions and progressively, stroke survivors are becoming more community-focused and less dependent on hospital-based care. This is proven as within five years, the frequency of patients who received home care increased significantly compared to those who were institutionalised (23, 26, 27).

Nur et al. (27), in the systematic review paper on the effectiveness of home-based care interventions for stroke survivors, have listed the six main factors influencing the need for home care, which are: i) increased life expectancy of the larger elderly population which increasingly affect the cost of public services; ii) changes in social status, values, and behaviour as in term of unavailability of children from taking care of the patients and also higher employment opportunities for women, which have reduced the time that they can dedicate to home care; iii) change in epidemiological trends that includes ability of non-communicable diseases to be treated at home; iv) improved economic condition, where treatment tools and equipment can be affordably own; v) changes in attitude and expectations as home care could be equipped with better equipment and technologies; and vi) changes in both policy and choice. The effectiveness of home care programmes has been shown with the reduction of hospitalisation time, thus optimising hospital bed usage, decreasing patient re-hospitalisation and constructively creating better connections among family members and caregivers, with more patient participation in activities for daily living (27).

When it comes to home-based stroke rehabilitation, caregivers play a very important role in supporting stroke survivors’ recovery and management. Caregivers are divided into formal caregivers and informal caregivers. A formal caregiver is a provider associated with a formal service system, whether a paid worker or a volunteer. While an informal (family or primary) caregiver is any relative, partner, friend or neighbour who has a significant personal relationship with and provides a broad range of assistance for an older person or an adult with a chronic or disabling condition, including stroke (28).

In Muhrodji et al. (29) research report, caregivers’ responsibilities are divided into three main themes: i) communication; ii) patients’ health; and iii) patients’ psychological aspects. Caregivers are expected to connect patients with medical personnel. Matters that need to be taken care of include patients’ medical appointments, support group involvement and personal inquiries regarding the patients. Caregivers also need to participate in maintaining patients’ health conditions by fulfilling basic needs and assisting rehabilitation, as well as maintaining patients’ psychological conditions by encouraging conversation, telling jokes, or recreation. Other important aspects that are on caregivers’ shoulders are financial responsibility and creating a safe environment for the patients (29, 30). The same report also mentions a few themes of problems caregivers need to face in order to manage stroke patients. Due to lack of knowledge and experience, caregivers are struggling to get information, services or help. Other than this, caregivers are dealing with personal emotional and physical limitations. It is reported that most caregivers feel underappreciated, experience major burnout, and often deal with uncooperative patients and families (30).

Wider recognition of the responsibilities of caregivers has given rise to an increase in the number of publications that address their concerns. A bibliometric analysis of stroke caregiver research has been done on the peer-reviewed journals published between 1989 and 2022 (10). It was reported that the co-occurrence keywords, including stroke survivors, burden, quality of life, depression, care and rehabilitation have revealed a broad spectrum of subjects in: i) understanding the needs and caregiving experiences; ii) the complications; and iii) targeted interventions for both stroke survivors and caregivers. It is also important to bring attention to the limitations and lack of publications in this field of research for low- and middle-income countries, where they were found to be more in need of a care support system for stroke caregivers (10).

As Table 2 summarises the stroke rehabilitation and management-related aspects identified as discussed, it is important to assist caregivers in getting access to stroke care, whether informational, communication, educational, support or others. Caregivers must be well-equipped to care for stroke patients and they need to be exposed to every issue, news, skill, and intervention that will help facilitate their roles.

### Stroke in Global Digital Health

Following the new generation of technologies, it is a convenient approach to provide stroke rehabilitation services not just in health facilities but also online. Throughout this emerging modern era of stroke research, a number of digital health interventions for stroke have been launched and applied. Globally, the initiative of digitising health services and expanding the sharing of information on health has taken off for quite some time already. Digital health is defined as the field of knowledge and practice associated with the development and use of digital technologies to improve health (31). It makes use of data and technology to: i) enhance patient outcomes and healthcare service delivery; ii) involve patients and encourage healthy lifestyle choices; and iii) create health data repositories to increase population-based research. The terms “telehealth,” “telemedicine,” “eHealth,” and “mHealth,” which all fall under digital health inclusions, are already common in the medical health field, especially in this digital, informational era (32). The terms “telehealth,” “telemedicine,” “eHealth,” and “mHealth,” all of which fall under the broader umbrella of digital health, are already well-established in the medical field, particularly in this digital and information-driven era.

Modern telemedicine has been in existence for approximately 50 years, since the late 1960s, by the National Aeronautics and Space Administration (NASA), for remotely monitoring the health of astronauts in space and projects (33). Telemedicine, which is defined as the remote diagnosis and treatment of patients by means of telecommunications technology, incorporates the use of technology and telecommunication systems to administer healthcare to patients who are geographically separated from providers. Telehealth, an extension of telemedicine, is defined as the delivery and facilitation of health and health-related services, including medical care, provider and patient education, health information services and self-care via telecommunications and digital communication technologies (34). The emergence of eHealth, defined by the WHO as the use of information and communication technologies (ICT) for health, works with partners at the global, regional, and country levels to promote and strengthen the use of ICT in health development, from applications in the field to the global governance (35). From there, several technologies have been introduced throughout the years, following the technology trend of progressive eras, including mHealth, video and audio technologies, digital photography, remote patient monitoring (RPM), and store and forward technologies (34). mHealth app is defined as software programmes designed to run on smartphones, tablets, or other portable devices that support health-related services, including monitoring, education, and rehabilitation for stroke patients (35).

As reported by the WHO Global Observatory for eHealth (GOe) (35), to support the achievement of health objectives and to transform the face of health service delivery across the globe, mobile and wireless technologies could be the driving force behind this change with their rapid advances and integration into existing eHealth services. mHealth involves the use and capitalisation of a mobile phone’s core utility of voice and short messaging service (SMS) as well as more complex functionalities and applications including the general packet radio service (GPRS), third and fourth generation mobile telecommunications (3G and 4G systems), global positioning system (GPS) and Bluetooth technology (35). Based on 2016 Global Health Observatory (GHO) (36) data by WHO, 109 out of 125 responding countries reported at least one mHealth programme in their country. A total of 80% of the responding low-income countries reported at least one mHealth programme, as compared to 91% of high-income countries. The number of programmes reported as being established has undergone rapid growth since 2010, yet only 16 countries have reported an evaluation of government-sponsored mHealth programmes (36). mHealth is primarily being applied in maternal and child health, and programmes reducing the burden of the diseases linked with poverty, including HIV/AIDS, malaria, and tuberculosis (35). Other than that, chronic diseases such as diabetes mellitus, cardiovascular disease, and chronic lung diseases, as well as mental health-related diseases, are also available (37).

The existing mHealth applications, as reported by Oi-Mean et al. (38) were mostly designed to raise stroke knowledge and awareness such as Think F.A.S.T (Australian Commission on Safety and Quality in Health Care) (39), Spot a Stroke FAST (American Heart Association and American Stroke Association) (40), the NIH Stroke Scale (National Institute of Health Stroke Scale Organization) (41), and Stroke 119 by Ministry of Health and Welfare, Republic of Korea (42). There is also Constant Therapy (Constant Therapy Health, Inc.), designed for people who have had a brain injury or cognitive disorder to give them direct access to clinical exercises that can rebuild their cognitive, speech, and language functions (43); the FAST-ED (Latest version app name: JoinTriage) by Allm Inc. (44), which focuses on the field assessment and destination triage of patients with acute ischaemic stroke; and AFib 2getherTM by Pfizer Inc. (45) that helps improve shared decision making discussions between patients with atrial fibrillation, their caregivers, and their provider, on the risk of stroke due to atrial fibrillation. In 2020, Neofect USA launched a paid mobile application, “Rehabit,” a lifestyle app for stroke survivors. “Rehabit” is a comprehensive wellness and stroke recovery programme, including recovery tips, lifestyle suggestions, video exercises, daily activities tracking and analysis, as well as extensive research on health and wellness reports (46). The most advanced mobile app reported is the FAST AI by Neuronics Medical (47), a fully automated smartphone application for the detection of severe stroke using machine learning algorithms to recognise facial asymmetry (drooping of the muscles in the face), arm weakness and speech changes. The app aims to help physicians manage their patients and test them for acute stroke symptoms, and was launched online on 31 October 2023. More information on the mentioned apps is included in Table 3.

A study by Dubey et al. (48) was done to evaluate the availability and content of stroke-related applications on the Apple iTunes and Android Google Play Store in 2013, and a total of 93 relevant applications were identified. Another quite similar study, but a more updated version by Piran et al. (49), on investigating available apps focused on stroke survivors and caregivers was reported. A systematic review of the medical apps in the US Apple iTunes Store available between 2013 and 2016 resulted in about 843 eligible apps being identified, with over 70 medical apps that existed to support stroke survivors and caregivers specifically. For China, the most current status of stroke-related smartphone applications available was about 145 apps obtained from six app stores (Android market, 360 app market, Wan Dou Jia, Ying Yong Bao and Apple app store) searched with keywords in Chinese and English, respectively, from June to August 2018. Further evaluation was done on 127 apps to determine the online health quality, and it was reported that the available apps were critically insufficient, hence the need for improvement in functionality and information quality (50).

All these reported findings prove that mHealth is no longer an unknown tool for medical services; however, the inclusivity and exposure of its usage are varied. Even with the availability of mHealth apps, the effectiveness of mHealth applications is still understudied and needs deeper research with a larger population.

### Stroke Intervention Modules for Web-Based and Smartphone Applications

In developing a proper digital health application, the procedure should always begin with the development of appropriate intervention modules. From the intervention procedure, the materials and applicable contents could be constructed and created. During this process, the involvement of multilevel and multivariant stakeholders could widen the context and framework for building up suitable, strategically designed intervention modules that can later be implemented for the target users. Some digital health intervention modules that have already been reported were developed with specific objectives according to diseases or health themes.

A research team in India successfully developed a web-based smartphone-enabled educational intervention for the management of stroke patients, “Care for Stroke,” in 2015 (51). The big research project started with a systematic review of India’s background on stroke incidence and prevalence, epidemiology (52) and the gaps in post-stroke rehabilitation, including from a social care perspective, physical and mental aspects, environmental barriers, health strategies and services (53), and many more issues being thoroughly discussed. With comprehensive information and backgrounds in hand, a proper protocol for a formative research study on the development of the intervention modules was constructed. The protocol of development and evaluation of smartphone-enabled, caregiver-supported educational intervention for the management of physical disabilities following stroke in India was divided into three phases: i) th development of the intervention; ii) pre-testing of the intervention and stakeholder consultation; and iii) piloting of the intervention, and assessment of feasibility and acceptability (54).

The “Care for Stroke” intervention contents were systematically developed to address the needs of stroke survivors in India. This culture-specific and language-specific intervention includes not only accessible information on stroke but also digitised audio-visual format contents from qualified stroke rehabilitation professionals in the home-based exercises, functional skills, guides, and assistive devices section (51). The outcomes of this smartphone-enabled intervention’s field testing revealed operational issues with connectivity, video streaming, visual clarity, video quality, and application functioning. Even so, after the intervention was reviewed, revised, and finalised for pilot testing, the results show that the “Care for Stroke” intervention was feasible and acceptable. Over 90% of the study participants found the intervention was relevant, comprehensible and useful. Over 96% of the stroke survivors and the caregivers rated the intervention as excellent and very useful. The results affirmed that the adoption and modification of this technology-based intervention could not only bridge the gaps in access to stroke rehabilitation services in India but also potentially in other low-resourced countries (53).

Similarly, Denham et al. (55) were also involved in developing an online secondary prevention programme for stroke survivors, “Prevent 2nd Stroke (PS2)”. This programme aims to address the modifiable health risk behaviours of stroke, which are blood pressure, physical activity, nutrition, depression and anxiety, smoking, and alcohol consumption. PS2 was developed as an eight-module online secondary. PS2 was modelled on the DoTTI (Design and develOpment, Testing early iterations, Testing for effectiveness, Integration and Implementation) framework for the development of online programmes. The programme has undergone four phases of development: i) content development and design; ii) testing early iteration; iii) testing for effectiveness; and iv) integration and implementation. This four-phase framework is evidence-informed and consumer-centred and could be applied widely to develop web-based programmes for a diverse range of diseases at each step. The stakeholders, which consist of researchers, clinicians, consumers, and programmers, are engaged in consultations to review progress and provide feedback on versions of the web-based tool. The inputs and feedback from the stakeholders are important to determine the appropriate next steps in development (56). From the PS2 intervention web-based intervention module study, it is reported that adult stroke survivors who completed a telephone survey and then participated in the online PS2 health programme and completed the six-month follow-up survey showed a betterment in their overall health and well-being compared to those who received generic health behaviour information. This proved that online platforms were a practical and impactful model to address the health information needs in stroke recovery and rehabilitation (55).

A primary-care-based integrated mHealth intervention, “SINEMA intervention” (57), is designed to address the system barriers by strengthening the capacity of the village doctors in rural China through training and support, and to shift the tasks of post-acute stage management from tertiary hospital to community-based primary healthcare settings for stroke survivors. This intervention undergone five stages of development, which involve: i) conducting literature review on existing message banks and analysing the characteristics of the banks; ii) interviewing stroke patients and caregivers to identify their needs; iii) drafting message contents and designing dispatching algorithms for a three-month pilot testing; iv) collecting feedback from pilot participants through questionnaire survey and in-depth interviews on facilitators and barriers related to their acceptance and understanding of messages; and v) finalising the message-based intervention based on participants’ feedback for the SINEMA trial (58). The stakeholders involved in SINEMA intervention include physicians at township and county hospitals, village doctors, stroke survivors and family caregivers. The SINEMA app contains: i) modules facilitating self-training on secondary prevention of stroke; ii) follow-up visit reminder setting; iii) follow-up visit guides; iv) data collection for the intervention part; v) performance indicator displays; vi) third-party voice message application; and vii) quality control function to monitor village doctors’ performance. Findings from this comprehensive research demonstrated that the SINEMA programme was well implemented in rural China. The app effectively implemented interactions between its human-delivered and technology-enabled components, thereby improving the participants’ health, bolstering doctor-patient relationships, and bettering the healthcare system in general (59).

An initiative to apply mHealth technology-centred care has been approached in Ghana, called “The Phone-Based Interventions under Nurse Guidance after Stroke (PINGS)” (60). This module was optimised for stroke survivors, and it has four components: i) home blood pressure self-monitoring; ii) mobile phone consultations led by nurses to address high domiciliary blood pressure readings of participants; iii) use of phone alerts as reminders to take medications; and iv) patient education via audio messages and interactive voice recordings. The PINGS initiative was regarded as feasible, acceptable, and appropriate by health workers in Ghana, as a high proportion agreed that the implementation of mHealth for post-stroke was implementable in resource-constrained settings (60, 61).

Another digital intervention for stroke patients, “The BP: Together,” used a person-based approach with integral patient and public involvement (62). Adapted from existing BP self-monitoring interventions (63), “The BP: Together” was delivered via mobile phone or web interface to support home self-monitoring of BP by sending out alerts to patients and their clinicians when average readings are above target to initiate planned medication changes. This intervention was designed in three phases: i) prototype intervention, by identifying materials, possible barriers to engagement and appropriate modification; ii) retrospective interviews with stroke patients to explore their experience and further identify barriers to engagement; and iii) feasibility study, to explore the concerns on the intervention implementation in primary care with focus groups of health care professionals. This intervention managed to demonstrate the integration of the person-based approach and contributed to the development of complex health interventions for a wider range of people (62).

Even though some of the intervention modules (Table 4) are not specifically for stroke, some of the contents and functions are related to stroke and are the basic determinable factor in monitoring health status. Yet, it is shown that there is somehow still no one definite protocol of content to be included in the modules. The inconsistency might be due to the different backgrounds of target users or limitations on the research parts or budgets, which are all challenges that need to be addressed for future work.

### Malaysia, Mobile Health (mHealth) and Stroke Intervention Module

In line with the advancement of technology and the increasing number of internet and smartphone users, it is appropriate for Malaysia to also keep up with the new era trend. Based on the Statista website report by J. Muller on smartphone users in Malaysia from 2015 to 2025, Malaysia had 28.98 million users in 2019 and is estimated to reach 30.41 million users in 2020 and is expected to reach over 33 million by 2024 (64). A feasible and accessible stroke intervention module smartphone-based application moderated for local patients, their caregivers and health professionals, as well as for the public, would be a great hand in proceeding with the current technology and health services quality.

Among the responding countries, Malaysia’s level of eHealth was reported to be on an advanced level compared to other countries, where most of the health services and information provided by mHealth programmes are established at the national level (35). A systematic review done on online medical mobile applications in Malaysia from 2014 to 2018 concluded that even mHealth apps are very common and the availability of internet with Android smartphones is growing day by day, it does need more improvement for this region (65). With the COVID-19 outbreak, it is expected that consumers will seek out alternative methods for health monitoring and consultation in an attempt to reduce their attendance at hospitals and clinics. As reported by GMO Research in 2020 (66), an online research platform, a survey conducted on the adoption and usage of health and wellness apps in five different countries across Asia: Korea, Japan, Indonesia, Thailand and Malaysia showed 100% of respondents in Malaysia are using certain health and wellness apps. The highest proportion out of the countries surveyed and the top three apps are: vi) COVID-19 related apps; ii) sport and fitness activity tracking apps; and iii) diet and nutrition apps, while the bottom three apps are: i) medical advice and patient community; ii) meditation; and iii) pregnancy related apps (66). All the databases and statistics for Malaysia show the nation’s ability and feasibility in following the trend to empower the usage of mHealth to the highest level.

The latest stroke intervention module launched and reported for Malaysia was in 2020, the Regional Emergency Stroke Quick-Response Network (RESQ) by the Ministry of Higher Education Malaysia and Universiti Putra Malaysia, supported by the World Federation of Neurology (WFN) and the Asian-Australasian Federation of Interventional and Therapeutic Neuroradiology (AAFITN) (67). This module focuses on neurologic intervention in delivering services for hyperacute stroke, specifically in medical thrombolysis and mechanical thrombectomy. The RESQ was designed based on the needs of the current situation in the country, and they aim to standardise and tighten the cooperation between the emergency medical services, the emergency department team and the RESQ in the stroke care units (67). Since this module is centred on hospital-based stroke management and services, further steps need to be taken to improve home-based care for stroke patients in digital form.

In 2014, a research team from Universiti Teknologi Petronas, Malaysia, did a study on mHealth awareness in the pre-detection of mild stroke symptoms in Malaysia. The team aimed to develop a mobile application that helps in the early detection of mild stroke syndrome, as well as to raise awareness and knowledge levels among society about stroke. The Stroke Pre-Detection (PD) mobile application was implemented on the Android platform and was the self-check assessment concept, consisting of three sections: i) conscious test, ii) mobility test, and iii) self-check questions. The empirical results show that the majority of respondents believe that the mobile stroke application can increase their stroke awareness and help them to perform early detection of mild stroke symptoms (38). Even though the study was just a prototype of mobile application, it is already suggesting how relevant and in need would be mobile stroke application usage by Malaysians, which currently has no reported stroke intervention mobile application available on the online market at that time.

A decade has passed, and yet there is still no official launch for stroke apps in the digital market for Malaysian-based users exclusively. However, due to COVID-19, the idea has once again emerged as the burden of stroke caregivers escalated and was affected greatly, which has made the urge to have such stroke mHealth into existence even more prominent this time. A qualitative exploration study reported on family caregivers’ experiences in Malaysia (68) highlighted that the pandemic and its implemented control measures had worsened the pre-existing issues involving social life, formal duty, and appointment schedules for stroke patients and caregivers. Hence, pinpointing that one of the strategies that could provide better assistance in the matter is to introduce, encourage, and emphasise the consistent use of telehealth in the healthcare system. Following that, another specific qualitative study on exploring the need for mobile application in stroke management by informal caregivers concluded that the respondents indeed believed that a stroke mobile application could help and act as a mediating tool to improve stroke patients’ quality of life and caregivers’ welfare on a daily basis for managing stroke in the long run (68).

Another in-depth study (7) has been conducted to explore the experiences of caregiving and access to stroke care from both caregivers and healthcare providers perspectives in Malaysia. In conjunction with the previous studies mentioned (68, 69), the respondents also suggested that an innovative intervention, such as the use of technology and advances in remote management of health, is needed to empower the caregivers of stroke survivors and improve the quality of stroke care, caregiving skills and competency. A mHealth application for stroke survivors and their caregivers would be able to address the unmet need for rehabilitation and close the gap Malaysia’s health system currently has (70). Malaysia’s healthcare providers accepted and supported the introduction of the mHealth app in assisting stroke caregivers; more than 80% responded with the intention to use it; more than 70% perceived that mHealth to be useful; and more than 60% expected it to be easy to use (if available) (7, 70).

**Figure 2:**
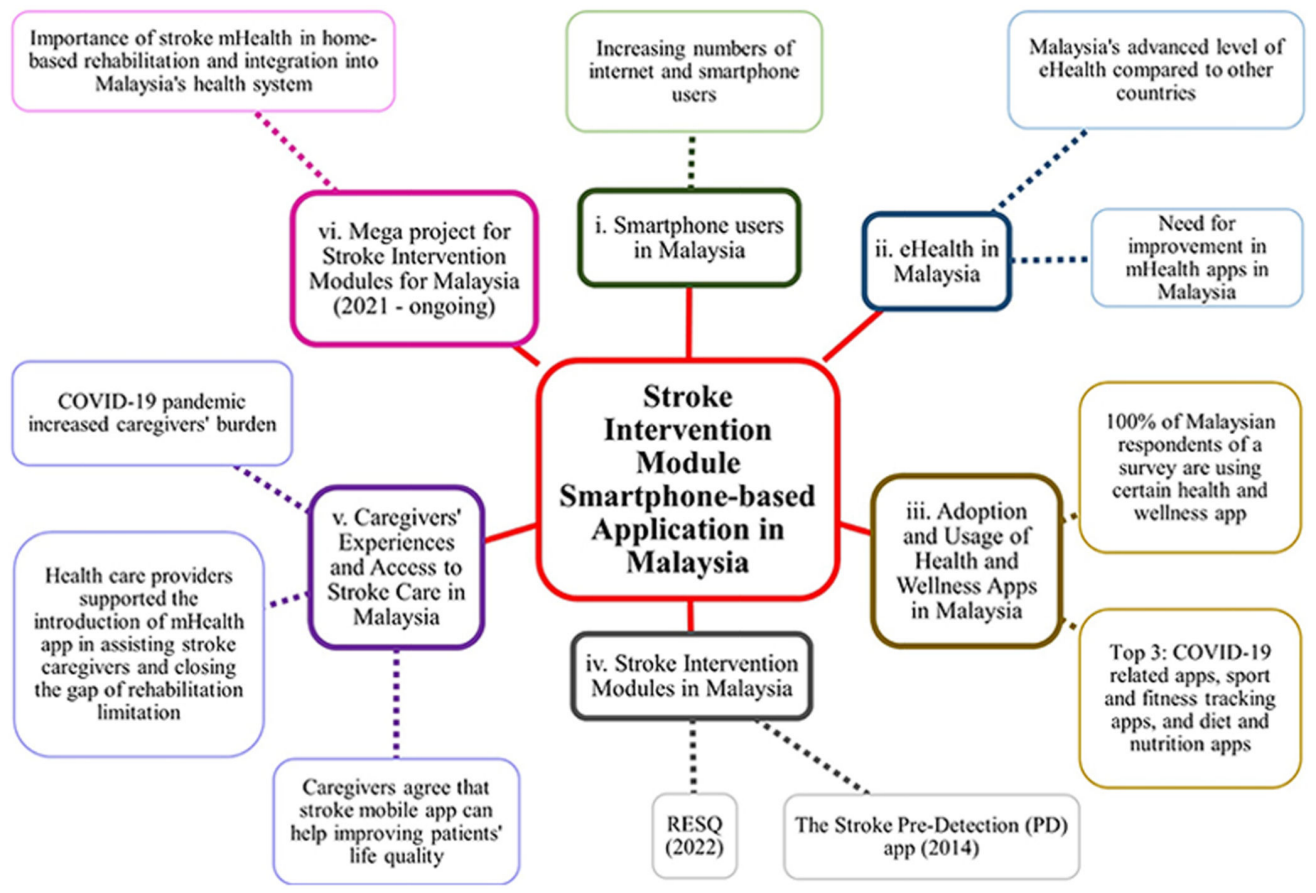
This mind map summarises the idea and discussion on the stroke intervention module smartphone-based application in Malaysia

Understanding this and realising the importance of having a mHealth application for stroke care, a mega project funded by the Newton-Ungku Omar Fund (2019) to develop intervention modules for stroke patients in Malaysia is currently ongoing by the collaborated research team from Universiti Sains Malaysia and the London School of Hygiene and Tropical Medicine (LSHTM), United Kingdom. The LSHTM team has previously successfully developed a smartphone-enabled intervention for stroke caregivers in India (51), and it has inspired the Malaysian team to extend the digital intervention concept. Stroke mHealth is expected to be a useful tool, not only to cater stroke caregivers for home-based rehabilitation but also to be integrated into Malaysia’s health system to create a better environment for the stroke community and health providers (Figure 2).

## Discussion

This narrative review has explored several aspects of stroke management and the associated burden on caregivers, with a particular focus on the role of digital health solutions globally and within Malaysia. The findings reveal the significant emotional, physical, and financial challenges faced by caregivers, compounded by the rising global burden of stroke, particularly in ageing populations (28, 29). These challenges underscore the urgent need for innovative, scalable interventions, especially in regions with limited healthcare infrastructure (3).

Digital health technologies have emerged as promising tools for stroke rehabilitation and caregiver support, particularly web-based and smartphone applications. These platforms extend care beyond clinical settings, enabling remote monitoring, personalised rehabilitation programmes, and continuous education for both stroke survivors and caregivers (37, 49). Globally, such technologies have demonstrated the potential to improve patient adherence to rehabilitation regimens, enhancing caregiver involvement and reducing the logistical challenges of traditional in-person care (48, 52). However, despite these advancements, challenges such as digital literacy, equitable access, and integration with existing healthcare systems remain significant obstacles, particularly in low- and middle-income countries (LMICs) (51, 52, 54, 71).

The integration of stroke intervention modules into digital platforms, such as smartphone applications, has demonstrated improvements in patient adherence to rehabilitation programmes and caregiver involvement (48, 51). The review highlights that stroke intervention modules integrated into digital platforms are most effective when tailored to the specific needs of users. Features such as personalised exercise plans, real-time feedback, and culturally appropriate educational resources have been linked to improved outcomes. However, global literature also points to the need for more robust clinical trials and long-term studies to establish the efficacy, scalability, and sustainability of these interventions (58).

While the global landscape of mHealth solutions for stroke care is expanding rapidly, Malaysia’s progress in this area remains limited. Existing studies on mHealth in Malaysia primarily focus on telerehabilitation and caregiver engagement, addressing barriers such as infrastructure constraints, digital literacy gaps, and regional disparities in healthcare access (65, 67). Notably, early implementations of mHealth modules in Malaysia show promise, particularly in providing cost-effective and accessible care to remote and underserved populations (64). However, the limited number of localised studies highlights the need for further research to adapt and optimise international findings to Malaysia’s unique context.

Several barriers hinder the direct adoption of international mHealth solutions in Malaysia. These include technological infrastructure gaps, cultural differences in healthcare practices, and variations in caregiver roles and expectations. For instance, while global reviews (12, 13) highlight the widespread adoption of stroke-specific mHealth applications in high-income countries, Malaysia faces challenges in achieving similar integration due to limited resources and inconsistent policies supporting digital health (65). Addressing these gaps requires a localised approach to mHealth development, one that considers Malaysia’s healthcare infrastructure, patient demographics, and socio-cultural factors.

Moreover, the necessity of a localised review is reinforced by the unique caregiving dynamics in Malaysia, where family caregivers play a central role in stroke management. Research suggests that mHealth applications designed for Malaysian users should prioritise features that support caregivers, such as culturally appropriate educational content, caregiver-focused support tools, and functionality that enhances collaboration with healthcare providers (67). This approach not only ensures relevance but also addresses gaps in the feasibility and acceptance of international mHealth solutions within the Malaysian context.

## Conclusion

In conclusion, while digital health technologies, particularly mHealth applications, offer promising opportunities for improving stroke rehabilitation and caregiver support, significant challenges remain. For Malaysia to bridge the gap with global trends, concerted efforts are needed to address technological, infrastructural, and accessibility barriers. Collaborative initiatives involving healthcare professionals, technologists, and policymakers will be critical to developing user-centred, evidence-based mHealth solutions tailored to Malaysia’s needs. Furthermore, ongoing evaluation and adaptation of these tools will be essential to ensure their long-term success and integration into the national healthcare system. By addressing these challenges, Malaysia can leverage digital health to transform stroke care, improving outcomes for patients and caregivers alike.

### Future Directions

The growing integration of digital health into everyday life is undeniable, as evidenced by the increasing number of mobile devices that come pre-loaded with health applications. This trend highlights not only the ubiquity of digital health but also the need for greater emphasis on refining and expanding its use. As digital health tools become more accessible, it is essential to ensure that their content is both relevant and effective. Further research is required to assess the quality, accessibility, and feasibility of these digital health interventions, particularly regarding stroke care. Well-designed digital health modules have the potential to empower individuals with accurate health information and support self-monitoring and management, all while benefiting from professional oversight.

Globally, remote care technologies, including mHealth applications, have proven their potential to bridge gaps in healthcare delivery, especially for chronic conditions like stroke. These technologies enable continuous care, provide access to rehabilitation resources, and reduce the burden on traditional healthcare systems. By integrating remote care technologies into routine stroke management, healthcare systems can enhance accessibility and efficiency. This is particularly critical for countries like Malaysia, where the availability of stroke-specific mHealth applications remains limited.

Malaysia, in particular, has lagged behind in the availability and development of stroke-specific mHealth applications. As other nations advance in integrating digital health into their healthcare systems, Malaysia must prioritise catching up to enhance its health services on the global stage. The growing demand for such technologies raises an important question: Does Malaysia possess the necessary resources, expertise, and determination to develop an effective stroke mHealth app that can be seamlessly integrated into its health system? With the encouraging global trends and local statistics, the potential is there, and addressing these gaps is key to shaping the future of stroke care in the country.

Future efforts should focus on enhancing collaboration between healthcare professionals, technologists, and policymakers to create user-centred, evidence-based applications. Additionally, ongoing evaluation and adaptation of these tools will be critical to ensure their long-term success and integration into national health systems. By emphasising the necessity and adoption of remote care technologies within the broader context of digital health, this review contributes to a forward-looking perspective on stroke care advancements.

## Supplementary Material

Appendix

## Figures and Tables

**Table 1:** The list of keywords for search engine

Main keywords	Keywords inclusion
Stroke	Stroke, stroke care, stroke rehabilitation, stroke caregivers, home-based rehabilitation, stroke intervention
mHealth	Digital health, telehealth, Telemedicine, mobile health application, eHealth, online health, stroke mHealth
Malaysia	Other countries as references

**Table 2:** Stroke rehabilitation and management-related aspects identified

Stroke rehabilitation	
Team effort	Patients Family/Friends Medical teams Rehabilitation and therapists’ Team Social workers Others
Post-stroke therapy and recovery	Ongoing rehabilitation after leaving the hospital Facility-based outpatient services In-home rehabilitation services
Optimal stroke rehabilitation programme	Based on stroke severity Severe stroke patients; in-hospital intensive interdisciplinary stroke rehabilitation Moderate stroke: hospital programmes with comprehensive therapy and medical coverage Mild stroke: community-based rehabilitation, including home care
Advantages of home care rehabilitation	More involvement of family members or caregivers Utilisation of existing community resources Increased patient and family participation in healthcare
Community-focused stroke rehabilitation	Prevention, delay and management of stroke in the community Decreased dependence on hospital-based care Increased frequency of home care compared to institutionalised care
Factors influencing the need for home care	High cost of public services Unavailability of caregivers Change in epidemiological trends Affordable treatment tools and equipment Change in attitude and expectations Policy and choices
Effectiveness of home care programme	Reduction of hospitalisation time Decrease in patient re-hospitalisation Better connection among family members and caregivers Increased participation in ADL
Caregivers in home-based rehabilitation	Formal caregivers Informal caregivers
Caregivers’ responsibilities	Communication between patients and medical personnel Patients’ health and conditions, physical and psychological Financial and emotional aspects
Challenges faced by caregivers	Lack of knowledge and experience Emotional and physical limitations Feeling underappreciated and experiencing burnout Dealing with uncooperative patients and family
Research on stroke caregivers	Understanding needs and caregiving experience Complications and targeted interventions Limited research in low-income and middle-income countries
Supporting caregivers in home-based rehabilitation	Access to stroke care information, communication, education and support Equipping caregivers with the necessary skills and interventions

**Table 3:** Information on some available stroke-related apps

mHealth app	Developer (country origin, year)	Main contents/components	^a^Availability
Think F.A.S.T	Australian Commission on Safety and Quality in Health Care (Australia, 2013)	Recognise the warning symptoms and locate Australian hospitals with specialised stroke units in times of emergencies Providing tips on stroke, recognising the signs of stroke in plain text basis, quiz to improve the understanding on stroke symptoms Locate the nearest Stroke Emergency Care Centre	No
Spot a Stroke FAST	American Heart Association and American Stroke Association (USA, 2013)	Educate the society on stroke symptoms Consists of tips and resources on stroke Recognises the signs of stroke in plain text basis Video clips on fast detection of stroke symptoms	No
NIH Stroke Scale	Gumption Multimedia (USA, 2013)	Implementation of the NIH Stroke Scale Provide an easy to access reminder and calculator for bedside evaluation of a patient	Yes
Stroke 119	Yonsei Stroke Team (South Korea, 2014)	Indicate the current position and the nearest hospital, each hospital contacts, possible treatments Cartoon figures for facial palsy, arm weakness and slurred speech Simple information for diagnosis and emergency stroke guide	Yes
Constant Therapy	Constant Therapy Health, Inc. (USA, 2014)	Customised and ever-adjusting exercises based on users’ unique needs Tackle memory challenges, enhance communication skills, and regain everyday abilities through an individualised programme Monitor your progress with real-time, easy-to-understand performance dashboard Live customer support	Yes
FAST-ED (JoinTriage)	Allm Inc. (USA, 2017)	Assessments based on standard scales Informational purposes Provides instant and accurate triage using clinically proven algorithms Also assists rapid patient transport by suggesting medical institutions to the paramedics based on distance and required treatment	Yes
AFib 2gether™	Pfizer Inc. (USA, 2018)	Assist the patient-provider interaction and help facilitate a discussion about stroke risk Educational and informational content, including videos, useful links, etc.	Yes
Rehabit	Neofect USA (USA, 2019)	Provides customised habit tracker feature to track and adopt healthy wellness habits into users’ lifestyles Daily articles and educational material Daily video tutorials and professional exercise demonstrations Tailored exercised contents Record daily activities and mood	Yes
FAST AI	Neuronics Medical (USA, 2023)	Detecting patterns of acute stroke including facial asymmetry, speech changes, and arm weakness Help physicians manage their patients and test them for acute stroke symptoms	Yes

a Availability on Apple Store and/or Google Play as searched by 9 March 2024

**Table 4:** Examples of digital health interventions

Module title	Author (year)	Main contents/components	Development phases
Care for Stroke	Kamalakannan et al. (2015) (51)	Web-based smartphone-enabled educational intervention for stroke management Comprehensive information and backgrounds Culture-specific and language-specific intervention for India Digitised audio-visual format contents	The development of the intervention Pre-testing of the intervention and stakeholder consultation Piloting of the intervention, and assessment of feasibility and acceptability
Prevent 2nd Stroke (PS2)	Denham et al. (2019) (55)	A web-based intervention module Developed as an eight-module online secondary prevention programme for stroke survivors Addressing modifiable health risk behaviours of stroke	Content development and design Testing early iteration Testing for effectiveness Integration and implementation
SINEMA	Gong et al. (2019) (57)	Primary-care-based integrated mobile health intervention for rural China Modules facilitating self-training on secondary prevention of stroke Follow-up visits reminder setting Follow-up visit guides Data collection for the intervention part Performance indicator displays Third-party voice message application Quality control function to monitor village doctors’ performance	Conducting a literature review on existing message banks and analysing the characteristics of these banks Interviewing stroke patients and caregivers to identify their needs Drafting message contents and designing dispatching algorithms for a 3-month pilot testing Collecting feedback from pilot participants through a questionnaire survey and in-depth interviews on facilitators and barriers related to their acceptance and understanding of messages Finalising feedback for the SINEMA trial
PINGS	Amuasi et al. (2022) (60)	mHealth module optimised for stroke survivors in Ghana Home blood pressure self-monitoring Mobile phone consultations led by nurses Use of phone alerts as reminders to take medications Patient education via audio messages and interactive voice recordings	Not applicable
The BP: Together	Band et al. (2016) (63) Rai et al. (2021) (62)	Mobile phone and web interface To support home self-monitoring of blood pressure Send out alerts to patients and clinicians Integration of the person-based approach	Prototype intervention Retrospective interviews with stroke patients Feasibility study
