# Supplementary material for: A Narrative Review on Mobile Health (mHealth) App for Stroke Care and Rehabilitation Intervention for Malaysia
Source: Malays J Med Sci. Author manuscript; Available in PMC 2025 Jul 23. (PMC7617935; doi:10.21315/mjms-03-2025-154)
Supplement: Supplementary file 1 [file EMS206746-supplement-Appendix.pdf]

68. Haji Mukhti MI, Ibrahim MI, Tengku Ismail TA, Nadal IP, Kamalakannan S, Kinra S, et al. Exploring the need for mobile application in stroke management by informal caregivers: a qualitative study. *Int J Environ Res Public Health*. 2022;**19(19)**:12959. https://doi.org/10.3390/ijerph191912959

69. Haji Mukhti MI, Ibrahim MI, Tengku Ismail TA, Nadal IP, Kamalakannan S, Kinra S, et al. Family caregivers' experiences and coping strategies in managing stroke patients during the COVID-19 pandemic: a qualitative exploration study. *Int J Environ Res Public Health*. 2022;**19(2)**:942. https://doi.org/10.3390/ijerph19020942

70. Sidek NN, Tengku Ismail TA, Kamalakannan S, Chen XW, Romli MH, Mat Said MZ, et al. A mixed-methods study on the implementation of a mobile health application (mHealth app) for stroke caregivers in Malaysia: healthcare providers' perspective. *Front Neurol*. 2023;**14**:1222260. https://doi.org/10.3389/fneur.2023.1222260

71. Niyomyart A, Ruksakulpiwat S, Benjasirisan C, Phianhasin L, Nigussie K, Thorngthip S, et al. Current status of barriers to mHealth access among patients with stroke and steps toward the digital health era: systematic review. *JMIR Mhealth Uhealth*. 2024;**12**:e54511. https://doi.org/10.2196/54511

Appendix 1

This review article has been orally presented in the 1st Congress of Medicine and Health 2024, organised by Faculty of Medicine, Universiti Kebangsaan Malaysia on 22–23 June 2023.

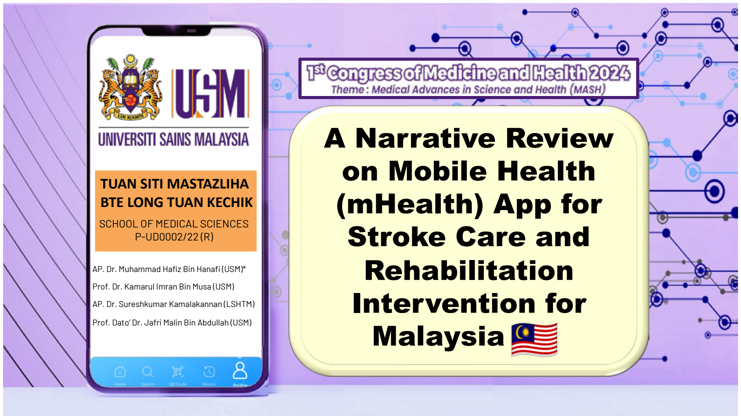

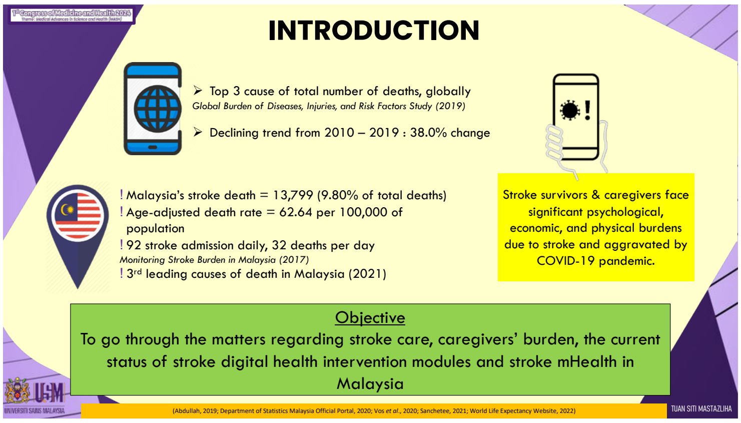

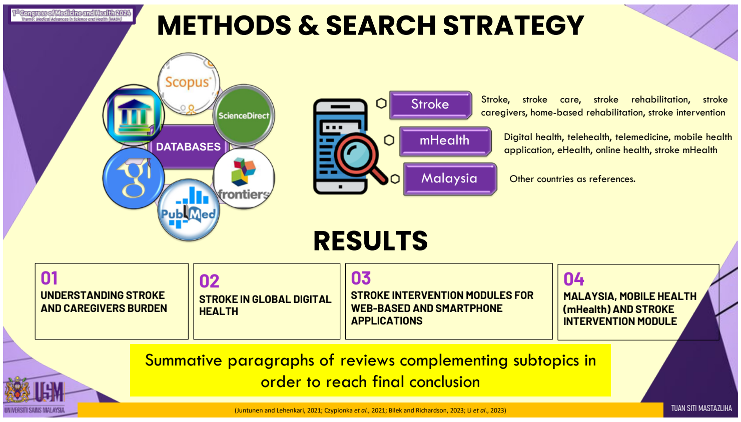
METHODS & SEARCH STRATEGY
Scopus
ScienceDirect
DATABASES
frontiers
PubMed
Stroke
Stroke, stroke care, stroke rehabilitation, stroke caregivers, home-based rehabilitation, stroke intervention
mHealth
Digital health, telehealth, telemedicine, mobile health application, eHealth, online health, stroke mHealth
Malaysia
Other countries as references.
RESULTS
01 UNDERSTANDING STROKE AND CAREGIVERS BURDEN
02 STROKE IN GLOBAL DIGITAL HEALTH
03 STROKE INTERVENTION MODULES FOR WEB-BASED AND SMARTPHONE APPLICATIONS
04 MALAYSIA, MOBILE HEALTH (mHealth) AND STROKE INTERVENTION MODULE
Summative paragraphs of reviews complementing subtopics in order to reach final conclusion
(Juntunen and Lehenkari, 2021; Czypionka et al., 2021; Bilek and Richardson, 2023; Li et al., 2023)
TUAN SITI MASTAZLIHA

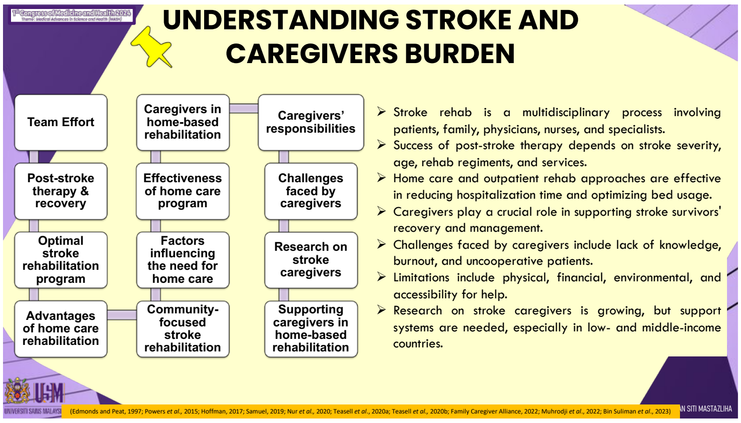
UNDERSTANDING STROKE AND CAREGIVERS BURDEN
Team Effort
Caregivers in home-based rehabilitation
Caregivers' responsibilities
Post-stroke therapy & recovery
Effectiveness of home care program
Challenges faced by caregivers
Optimal stroke rehabilitation program
Factors influencing the need for home care
Research on stroke caregivers
Advantages of home care rehabilitation
Community-focused stroke rehabilitation
Supporting caregivers in home-based rehabilitation
Stroke rehab is a multidisciplinary process involving patients, family, physicians, nurses, and specialists.
Success of post-stroke therapy depends on stroke severity, age, rehab regiments, and services.
Home care and outpatient rehab approaches are effective in reducing hospitalization time and optimizing bed usage.
Caregivers play a crucial role in supporting stroke survivors' recovery and management.
Challenges faced by caregivers include lack of knowledge, burnout, and uncooperative patients.
Limitations include physical, financial, environmental, and accessibility for help.
Research on stroke caregivers is growing, but support systems are needed, especially in low- and middle-income countries.
(Edmonds and Peat, 1997; Powers et al., 2015; Hoffman, 2017; Samuel, 2019; Nur et al., 2020; Teasell et al., 2020a; Teasell et al., 2020b; Family Caregiver Alliance, 2022; Muhrodji et al., 2022; Bin Suliman et al., 2023)

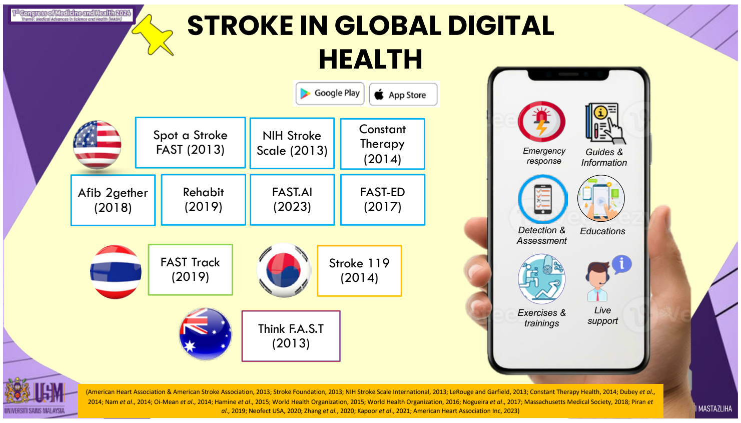
STROKE IN GLOBAL DIGITAL HEALTH
Google Play
App Store
Spot a Stroke FAST (2013)
NIH Stroke Scale (2013)
Constant Therapy (2014)
Afib 2gether (2018)
Rehabit (2019)
FAST.AI (2023)
FAST-ED (2017)
FAST Track (2019)
Stroke 119 (2014)
Think F.A.S.T (2013)
Emergency response
Guides & Information
Detection & Assessment
Educations
Exercises & trainings
Live support
(American Heart Association & American Stroke Association, 2013; Stroke Foundation, 2013; NIH Stroke Scale International, 2013; LeRouge and Garfield, 2013; Constant Therapy Health, 2014; Dubey et al., 2014; Nam et al., 2014; Oi-Mean et al., 2014; Hamine et al., 2015; World Health Organization, 2015; World Health Organization, 2016; Nogueira et al., 2017; Massachusetts Medical Society, 2018; Piran et al., 2019; Neofect USA, 2020; Zhang et al., 2020; Kapoor et al., 2021; American Heart Association Inc, 2023)

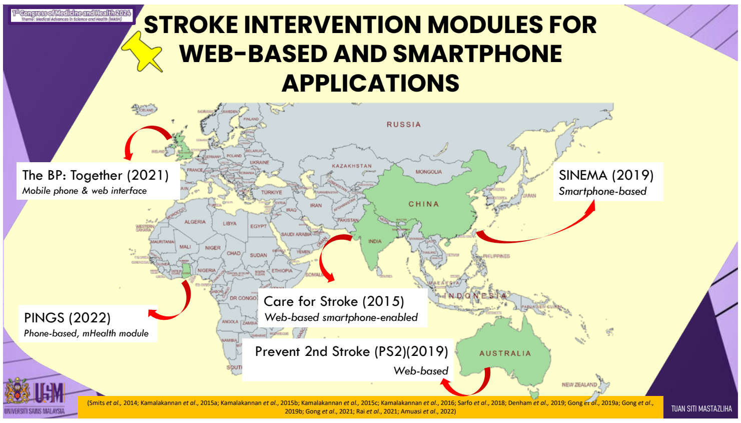
STROKE INTERVENTION MODULES FOR WEB-BASED AND SMARTPHONE APPLICATIONS
RUSSIA
CHINA
INDIA
INDONESIA
AUSTRALIA
NEW ZEALAND
The BP: Together (2021)
Mobile phone & web interface
SINEMA (2019)
Smartphone-based
Care for Stroke (2015)
Web-based smartphone-enabled
PINGS (2022)
Phone-based, mHealth module
Prevent 2nd Stroke (PS2)(2019)
Web-based
(Smits et al., 2014; Kamalakannan et al., 2015a; Kamalakannan et al., 2015b; Kamalakannan et al., 2015c; Kamalakannan et al., 2016; Sarfo et al., 2018; Denham et al., 2019; Gong et al., 2019a; Gong et al., 2019b; Gong et al., 2021; Rai et al., 2021; Amuasi et al., 2022)
TUAN SITI MASTAZLIHA

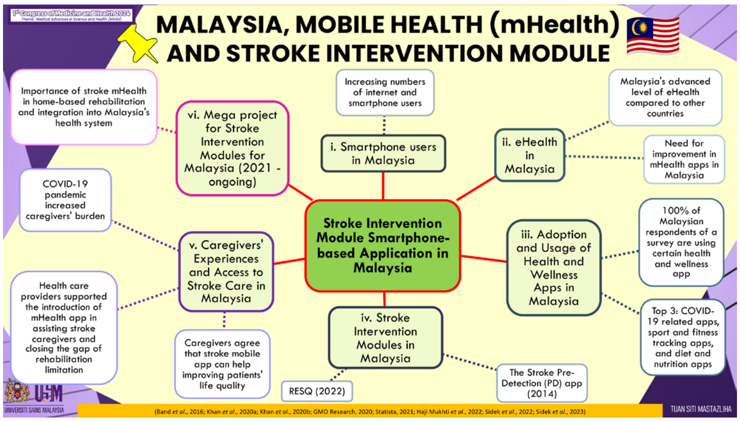
1st Congress of Medicine and Health 2024
Theme: Medical Advances in Science and Health (MASH)
MALAYSIA, MOBILE HEALTH (mHealth) AND STROKE INTERVENTION MODULE
Stroke Intervention Module Smartphone-based Application in Malaysia
i. Smartphone users in Malaysia
Increasing numbers of internet and smartphone users
ii. eHealth in Malaysia
Malaysia's advanced level of eHealth compared to other countries
Need for improvement in mHealth apps in Malaysia
iii. Adoption and Usage of Health and Wellness Apps in Malaysia
100% of Malaysian respondents of a survey are using certain health and wellness app
Top 3: COVID-19 related apps, sport and fitness tracking apps, and diet and nutrition apps
iv. Stroke Intervention Modules in Malaysia
RESQ (2022)
The Stroke Pre-Detection (PD) app (2014)
v. Caregivers' Experiences and Access to Stroke Care in Malaysia
COVID-19 pandemic increased caregivers' burden
Health care providers supported the introduction of mHealth app in assisting stroke caregivers and closing the gap of rehabilitation limitation
Caregivers agree that stroke mobile app can help improving patients' life quality
vi. Mega project for Stroke Intervention Modules for Malaysia (2021 - ongoing)
Importance of stroke mHealth in home-based rehabilitation and integration into Malaysia's health system
USM UNIVERSITI SAINS MALAYSIA
(Band et al., 2016; Khan et al., 2020a; Khan et al., 2020b; GMO Research, 2020; Statista, 2021; Haji Mukhti et al., 2022; Sidek et al., 2022; Sidek et al., 2023)
TUAN SITI MASTAZLIHA

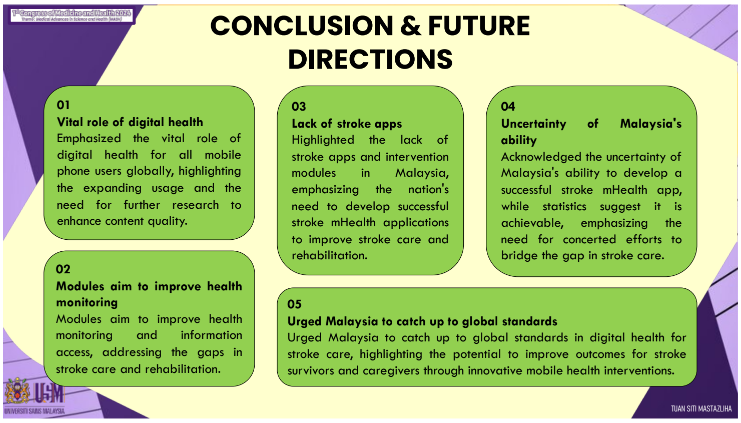
1st Congress of Medicine and Health 2024
Theme: Medical Advances in Science and Health (MASH)
CONCLUSION & FUTURE DIRECTIONS
01
Vital role of digital health
Emphasized the vital role of digital health for all mobile phone users globally, highlighting the expanding usage and the need for further research to enhance content quality.
02
Modules aim to improve health monitoring
Modules aim to improve health monitoring and information access, addressing the gaps in stroke care and rehabilitation.
03
Lack of stroke apps
Highlighted the lack of stroke apps and intervention modules in Malaysia, emphasizing the nation's need to develop successful stroke mHealth applications to improve stroke care and rehabilitation.
04
Uncertainty of Malaysia's ability
Acknowledged the uncertainty of Malaysia's ability to develop a successful stroke mHealth app, while statistics suggest it is achievable, emphasizing the need for concerted efforts to bridge the gap in stroke care.
05
Urged Malaysia to catch up to global standards
Urged Malaysia to catch up to global standards in digital health for stroke care, highlighting the potential to improve outcomes for stroke survivors and caregivers through innovative mobile health interventions.
USM UNIVERSITI SAINS MALAYSIA
TUAN SITI MASTAZLIHA

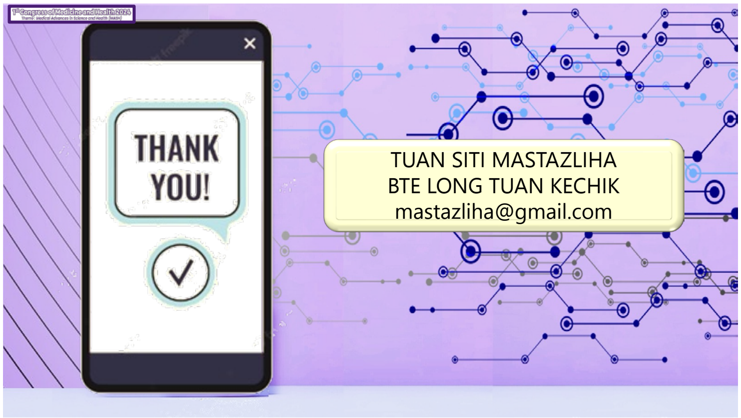
1st Congress of Medicine and Health 2024
THANK YOU!
TUAN SITI MASTAZLIHA
BTE LONG TUAN KECHIK
mastazliha@gmail.com
